# Primary stability of short stem prostheses for the shoulder—a biomechanical comparative study of two short stem designs

**DOI:** 10.1007/s00402-025-06041-1

**Published:** 2025-08-20

**Authors:** Anna-Katharina Nolte, Maxime Marie Seifert, Sebastian Jäger, J. Philippe Kretzer, Mareike Schonhoff, Felix Zeifang, Raphael Trefzer, Benjamin Panzram, Tobias Renkawitz, Matthias Bülhoff

**Affiliations:** 1 Department of Orthopaedics, University Hospital Heidelberg, Heidelberg, Germany; 2 Department of Orthopaedics Section of Biomechanics and Implant Research, University Hospital Heidelberg, Heidelberg, Germany; 3 Ethianum Klinik Heidelberg, Heidelberg, Germany; 4 Department of Orthopaedics, University Hospital Regensburg, Regensburg, Germany

**Keywords:** Short stem prosthesis, Primary stability, In-vitro, Biomechanics

## Abstract

**Introduction:**

The use of short stems has been a trend in shoulder arthroplasty within the last 15 years with excellent short- to mid-term clinical outcomes. Short stems anchor in the cancellous metaphysis and a high level of postoperative stability with absence of micro-movements at the bone-implant interface can be crucial for the following healing. The study aimed to assess the primary rotational stability of two uncemented short stems. The hypothesis was that the rotational stability is independent of implant design.

**Materials and methods:**

A biomechanical in-vitro study was conducted on 12 fresh-frozen (six matched pairs) human cadaveric shoulders. Two short stems, one with a cylindrical and one with a rectangular stem shape design, were implanted in a cement-free technique. A sinusoidal torque force was applied, starting from 0.5 Nm (load level one) to 3.0 Nm (load level six) with increasing load levels (0.5 Nm per 500 cycles). The relative rotation between stem and bone was assessed with an optical measurement system.

**Results:**

Mean donor age was 80 years (range 67–89 years), mean bone density was 0.41 g/cm^2^ (range 0.21–0.64 g/cm^2^) with no significant difference between stem design groups (*p* = 0.155). The cylindrical stem design demonstrated a significantly higher relative rotation than the rectangular stem design at 2.0 Nm (*p* = 0.047), 2.5 Nm (*p* = 0.034) and 3.0 Nm (*p* = 0.016). The metaphyseal and diaphyseal filling ratio was significantly higher in the rectangular stem design group (*p* = 0.002, *p* = 0.001).

**Conclusions:**

The cylindrical stem design shows a higher relative rotation in vitro imitating the immediate postoperative situation, indicating that rotational stability might depend on the implant design. However, the mid- to first long-term rates of aseptic stem loosening for the cylindrical stem design are generally low. It is important to consider the sensible postoperative healing phase during postoperative rehabilitation, especially for cylindrical stem designs, to promote secondary osseointegration.

**Level of evidence:**

Experimental study.

## Introduction

Short stem prostheses in shoulder arthroplasty offer the advantage of a bone-preserving implantation, making them advantageous in potential revision scenarios. Since Jost et al. [6] reported about the first case series of short stem prostheses for patients with primary osteoarthritis in 2011, numerous studies have demonstrated favorable short- to mid-term clinical outcomes [1, 7, 8, 20, 22], and low rates of aseptic stem loosening were reported in first mid- to long-term obervations [12]. However, a paucity of long-term clinical data remains and some concerns have emerged regarding the long-term osseointegration of short stem prostheses based on early radiological observations.

From a biomechanical standpoint, stable long-term osseointegration is achieved through (1) avoidance of stress shielding; (2) prevention of implant migration; and (3) high primary stability [23]. High rates of bone adaptions due to stress shielding, associated with high filling ratios and cortical contact [19], were observed in the first generation of short stems [17]. Eventually, design changes and implantation with reduced filling-ratio led to smaller rates of stress shielding over time [18]. Besides that, implant migration, also known as subsidence, has been demonstrated for short stems [4, 13, 21]. To this date, both stress shielding and subsidence have not been associated with inferior clinical outcomes, however, long-term observations are warranted [18, 21].

Primary stability is attained during the implantation process and a high level of primary stability with absence of relative micro-movements at the bone-implant interface is crucial for the following secondary healing process [23]. To our best knowledge, there is a paucity on biomechanical data for the primary stability of short stem prostheses and despite various short stem designs available on the market these days, the available clinical data predominantly derived from one design (Ascend Flex™, Stryker, Kalamazoo, MI, USA) [20, 22]. The study aimed to assess the primary rotational stability of two uncemented short stems with distinct designs. The hypothesis was that the rotational stability is independent of implant design.

## Methods

A biomechanical in-vitro study was conducted on 12 fresh-frozen (six matched pairs) human cadaveric shoulders (ScienceCare; Phoenix; AZ; USA). Ethical approval was obtained from the local Ethical Committee of the Medical Faculty (S-077/2022). Exclusion criteria were prior injury or surgery of the shoulder joint and a history of cancer. Bone density measuring was performed using the Dual X-Ray Absorptiometry (DXA) (Horizon Wi, Hologic, Marlborough, USA) method prior to randomization.

Preoperative anterior-posterior x-ray images were used for planning the size of the short stem prostheses (TraumaCad software, Brainlab AG, Munich, Germany). Specimen were then thawed pairwise at room temperature (23 °C) for approximately 24 h before soft tissue, scapula and clavicle were removed from each specimen.

### Study design

Each shoulder pair received two types of short stems with random allocation by means of a computer-generated list (Randlist 1.2; Datinf GmbH; Germany). The *Tornier Flex Shoulder System* (Stryker, Kalamazoo, MI, USA) with a cylindrical shape design (group A) and the *Medacta short stem prosthesis system* (Medacta, Castel San Pietro, Switzerland) with a rectangular shape design (group B) were each implanted in one of the two humeri of one donor in order to minimize donor-specific disruptive factors while comparing the two implants.

### Implants and surgical technique

Both shoulder systems were implanted by one highly experienced and certified shoulder surgeon. The characteristics of the implants are displayed in Table 1.

**Table 1:** Characteristics of the implants in group A and B. mm = millimeter

	Tornier Flex Shoulder System (group A) (Stryker, Kalamazoo, MI, USA)	Medacta short stem prosthesis system (group B) (Medacta, Castel San Pietro, Switzerland)
Stem length	66–94 mm	54.1–66.5* mm
Coating	PTC Titanium-Plasma	Hydroxylapatit
Geometry	Cylindrical shape design	Rectangular shape design

*without metaphysis

**Fig. 1:**
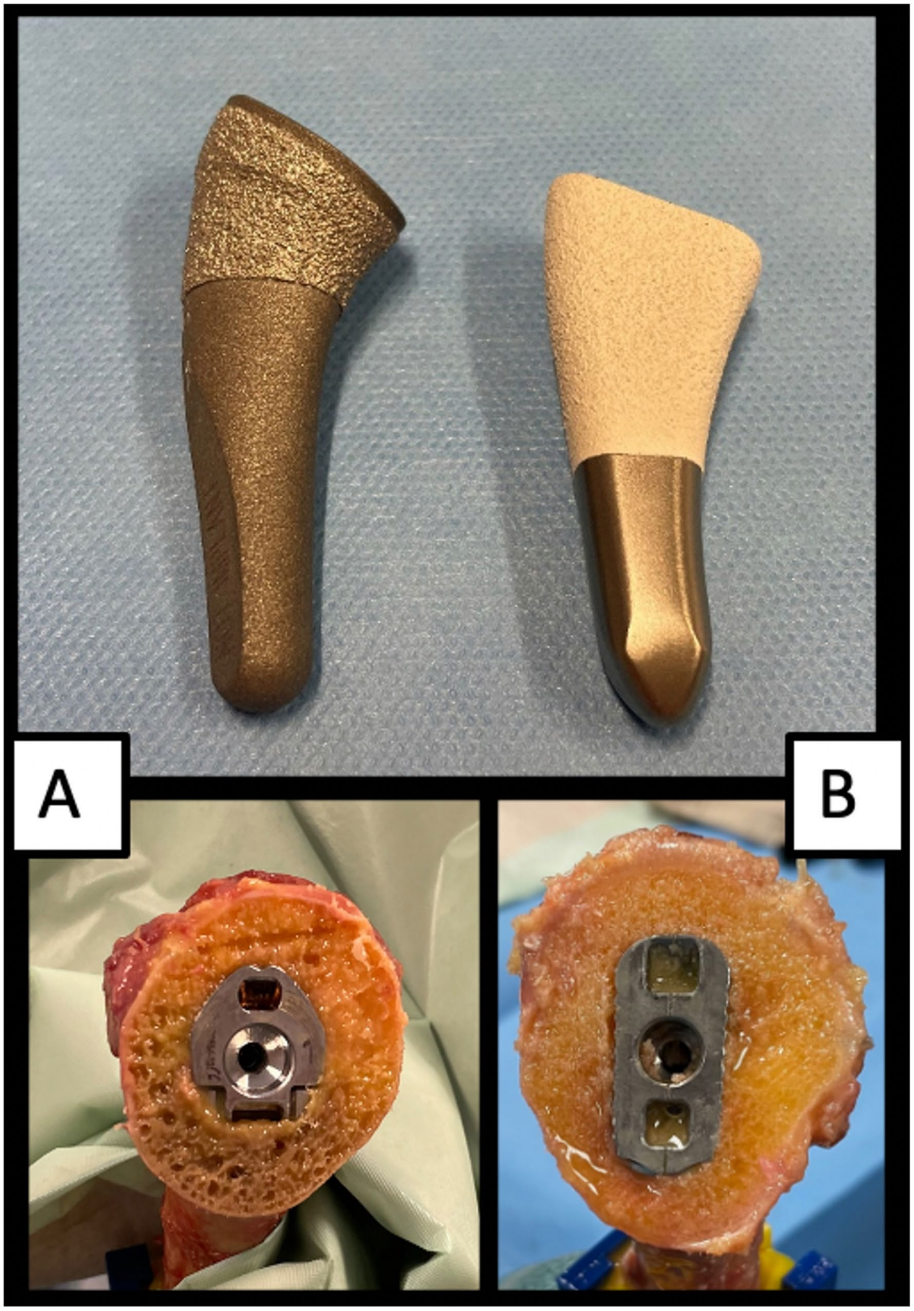
Demonstration of the two stem design types. **A** (Group A) *Tornier Flex Shoulder System* (Stryker, Kalamazoo, MI, USA) with a cylindric shape design. The stem is made of titanium, the distal portion of the stem is sandblasted and the bulky metaphysis is coated with a porous titanium coating (PTC) plasma spray. **B** (Group B) *Medacta short stem prosthesis* (Medacta, Castel San Pietro, Switzerland) with a rectangular shape design. The stem is made of titanium, the distal portion of the stem is sandblasted, the metaphysis is coated with a hydroxyapatite coating

Group A: The *Tornier Flex Shoulder System* (Stryker, Kalamazoo, MI, USA) is a modified version of the first short stem generation (Ascend™ Monolithic, Tornier Inc. ^®^). The system [11, 18] and implantation technique [16] have been described before in detail. The stem has a cylindric shape design, is made of titanium and while the distal portion of the stem is sandblasted, the bulky metaphysis is coated with a porous titanium coating (PTC) plasma spray (see Fig. 1). In theory, primary stabilization is achieved through the proximal stem portion that anchors in the compacted metaphyseal cancellous bone while the distal portion of the stem does not anchor in the bone but serves for shaft centering. The length of the humeral component varies between 66 and 94 mm (eight sizes). The inclination angle (neck-shaft angle) is determined by the stem, with options of 127.5°, 132.5°, or 137.5°. The prosthesis head, available in nine different diameters, various heights, and low or high offset, is connected to the stem via a tapered connection. The prostheses were then impacted in a cement-free technique.

Group B: The *Medacta short stem prosthesis* system (Medacta, Castel San Pietro, Switzerland) has a rectangular shape design. The stem is made of titanium, the distal portion of the stem is sandblasted and the metaphysis is coated with a hydroxyapatite coating (see Fig. 1). The length of the stem varies between 54.1 mm and 66.5 mm (eleven sizes). The inclination angle is determined by the metaphysis, with options of 128°, 135°, or 142°. Stem and metaphysis are connected through press-fit and a screw tightened with 6 Newtonmeter [Nm]. A double eccentric varies the offset between head and stem and the prosthesis head is available in ten different diameters.

First, the humeral head was resected with the help of a resection template and an oscillating saw. The medullary canal was then opened with an awl. Next, rasps of increasing sizes were utilized to compact the cancellous bone with the last used rasp determining the final stem size. After removing the rasp handles, the resection surfaces were adapted by a milling machine. On the back table, connections between stem and metaphysis were established and the prosthesis head was connected to the stem via a tapered connection. The prostheses were then impacted in a cement-free technique.

### Preparation for testing

The 12 humeri with the prostheses were degreased with isopropanol. In order to have equal torque distribution between each humeri, the implants were aligned horizontally before being casted in a two-component resin (RenCast FC 53 A/B, Goessl + Pfaff GmbH, Karlskron, Germany). An individual three-dimensional (3D) printed ring was attached to the head of the prosthesis with a two-component adhesive (UHU Plus Acrylit Express, Rotterdam, Netherlands).

Next, the humeri were integrated into a servo-hydraulic testing machine (MTS Mini Bionix 359, MTS System Corporation, Eden Prairie, Minnesota, USA). The resin block was secured with clamps, the rods of the 3D ring were clamped centrally between two vertical metal rods, which were attached to the testing head. To apply an axial load to the prosthesis, the testing head was then brought into contact with the implant head.

### Testing

The study aimed to simulate the early postoperative phase after implantation. The testing protocol was therefore developed according to the data by Bergmann et al. [3]. A sinusoidal torque force was transmitted to the prosthesis through the rods, starting at 0.5 Nm. Incrementally, the torque force was increased by 0.5 Nm after every 500 cycles, up to 3.0 Nm. This resulted in six load levels per specimen. To minimize potential differences in prosthesis impaction and prevent immediate proximal dislocation, a constant force of 50 N was applied to the head of the prosthesis.

Relative motion measurement between prosthesis and bone was conducted using an optical measurement system (PONTOS; GOM GmbH, Braunschweig, Germany), which recognizes optical markers through 3D triangulation. For this purpose, optical markers (GOM GmbH, Braunschweig, Germany) were placed at defined intervals along the stem axis, in the medial region of the surgical neck, and on the 3D ring. Before the torque was applied, baseline measurements were taken. Further measurements occurred at specific time points during each loading level, with measurements taken at the beginning of a loading level after 10 cycles, in the middle of a loading level after 250 cycles, and at the end of a loading level after 450 cycles. After the specimens had undergone six load levels, X-ray images in the anterior–posterior direction were taken.

### Data evaluation and statistics

To assess differences in bone quality between the two implant groups, a paired t-test was performed (SPSS Statistics Version 27, IBM, Armonk, USA), with a significance level set at *p* < 0.05.

**Fig. 2:**
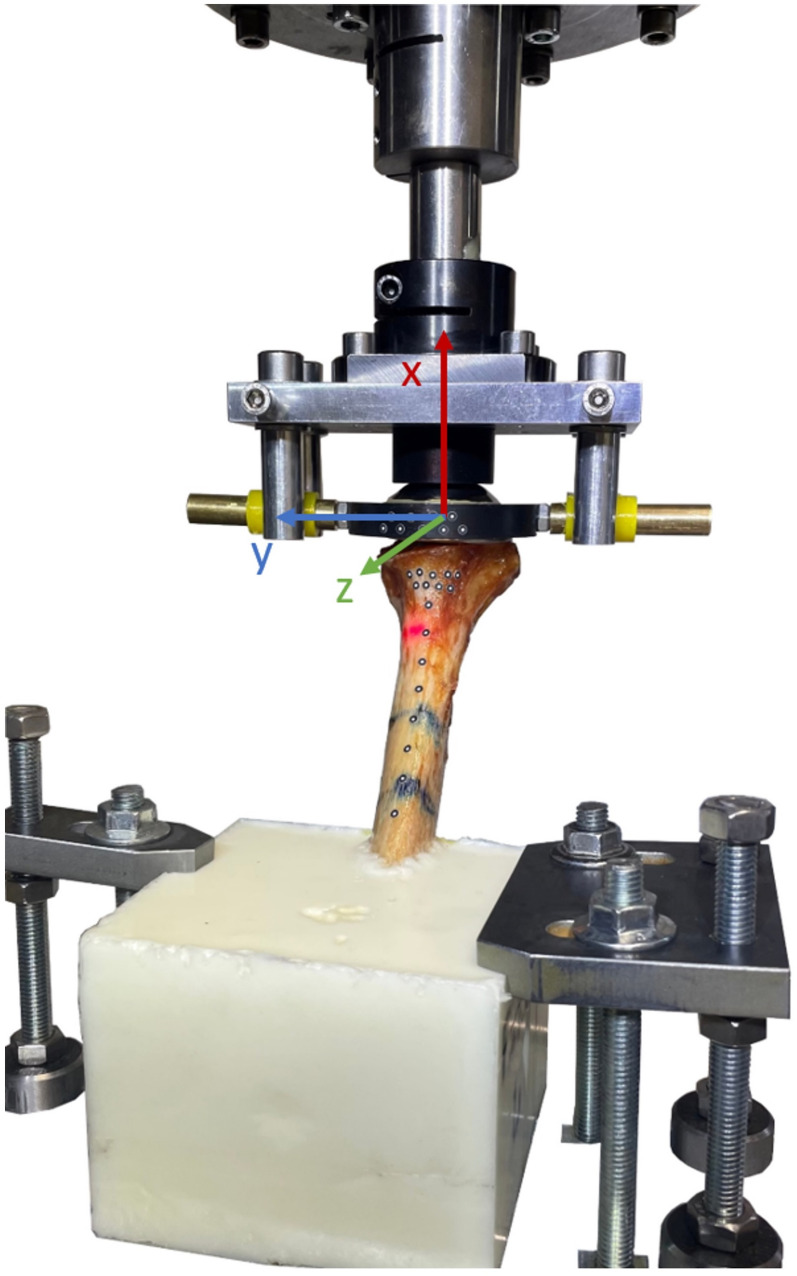
Coordinate system. x-axis: corresponding to the cone axis; y-axis: antero posterior direction; z-axis: oriented medially for right shoulders and laterally for left shoulders

The data were analyzed using the program associated with the measurement system (PONTOS; GOM GmbH, Braunschweig, Germany). Three components were defined: bone axis, humeral neck, and implant head. A coordinate system was created, with the x-axis corresponding to the cone axis, the y-axis in the antero–posterior direction, and the z-axis oriented medially for right shoulders and laterally for left shoulders (see Fig. 2). The values for each specimen were visualized in MATLAB (Version R2022a, MathWorks, Natick, USA). For each measurement, the arithmetic mean was determined, resulting in three rotation values per loading sequence and specimen. These were then used to calculate an arithmetic mean for each specimen and load level, which was used for statistical analysis. In addition to a detailed descriptive analysis, a statistical analysis was conducted at a significance level of *p* < 0.05. Cohen’s d was calculated to represent the effect size.

The metaphyseal and diaphyseal filling ratio of the prostheses was determined according to the method of Schnetzke et al. [17]. Therefore, X-ray images were imported into ImageJ (U.S. National Institutes of Health, Bethesda, Maryland, USA). For statistical analysis a paired t-test at a significance level of *p* < 0.05 was conducted.

## Results

### Specimen’s characteristics

The study collective comprised of four female and the two male donors. Mean donor age was 80 years (range 67–89), mean height 163 cm (range 147–180), mean weight 55.2 kg (range 37.6–68.0), and mean body-mass-index [BMI] 20.7 kg per square meter [kg/m^2^] (range 15.1–28.3). The average bone density for all specimens was 0.41 g/cm^2^ (range 0.21–0.64), with an average T-score of − 4.33 (range − 6 to − 2.6) (for demographics see Table 2). Specimen in the *Tornier Flex Shoulder System* group (group A) showed an average bone density of 0.43 g/cm^2^ (0.35–0.62), while the *Medacta short stem prosthesis* group (group B) had a mean bone density of 0.37 g/cm^2^ (range 0.21–0.64) without a significant statistical difference between groups (t(4) = − 1.753; *p* = 0.155; d = − 0.784).

**Table 2:** Demographics. Age, weight, height, BMI (= body mass index), BMD (= bone marrow density) for all specimen

Demographics	
Age	80 years (Range 67–89)
Weight	55.23 kg (Range 37.60–68.03)
Height	1.63 m (Range 1.47–1.80)
BMI	20.69 kg/m^2^ (Range 15.06–28.32)
BMD	0.41 g/cm^2^ (Range 0.21–0.64)

### Implant sizes

The sizes of the Tornier Ascend Flex models were as follows: three sizes 1, one each of sizes 2, 3, and 4. All had an inclination angle of 132.5°. The sizes of the Medacta models were as follows: four sizes 9, one size 10, and one size 13. The inclination angles were three 128° and three 135°.

### Testing results

One specimen was excluded from testing due to a periprosthetic fracture during implantation of the short stem. The following results are based on the data obtained from the remaining five intact shoulder pairs.

Tornier Flex Shoulder System (group A).

The rotation around the x axis increased with each load level. In this group, the increment between load level one and six was significant (t(4)= − 6.403; *p* = 0.003; d= − 2.864).

Medacta short stem prosthesis (group B).

The rotation around the x axis increased with each load level, however, the increment between load level one and six was not significant (t(4) = − 1.195; *p* = 0.298; d = − 0.534).

### Group assessment

There was no significant difference between the two groups in load level one (0.5 Nm) (t(4)=− 1.558; *p* = 0.194, d= − 0.697), two (1.0 Nm) (t(4)=− 1.872; *p* = 0.135; d= − 1.179) and three (1.5 Nm) (t(4)=− 2.635; *p* = 0.058; d=− 1.272), however, short stems of the *Tornier Flex Shoulder System* group (group A) showed more variability.

**Fig. 3:**
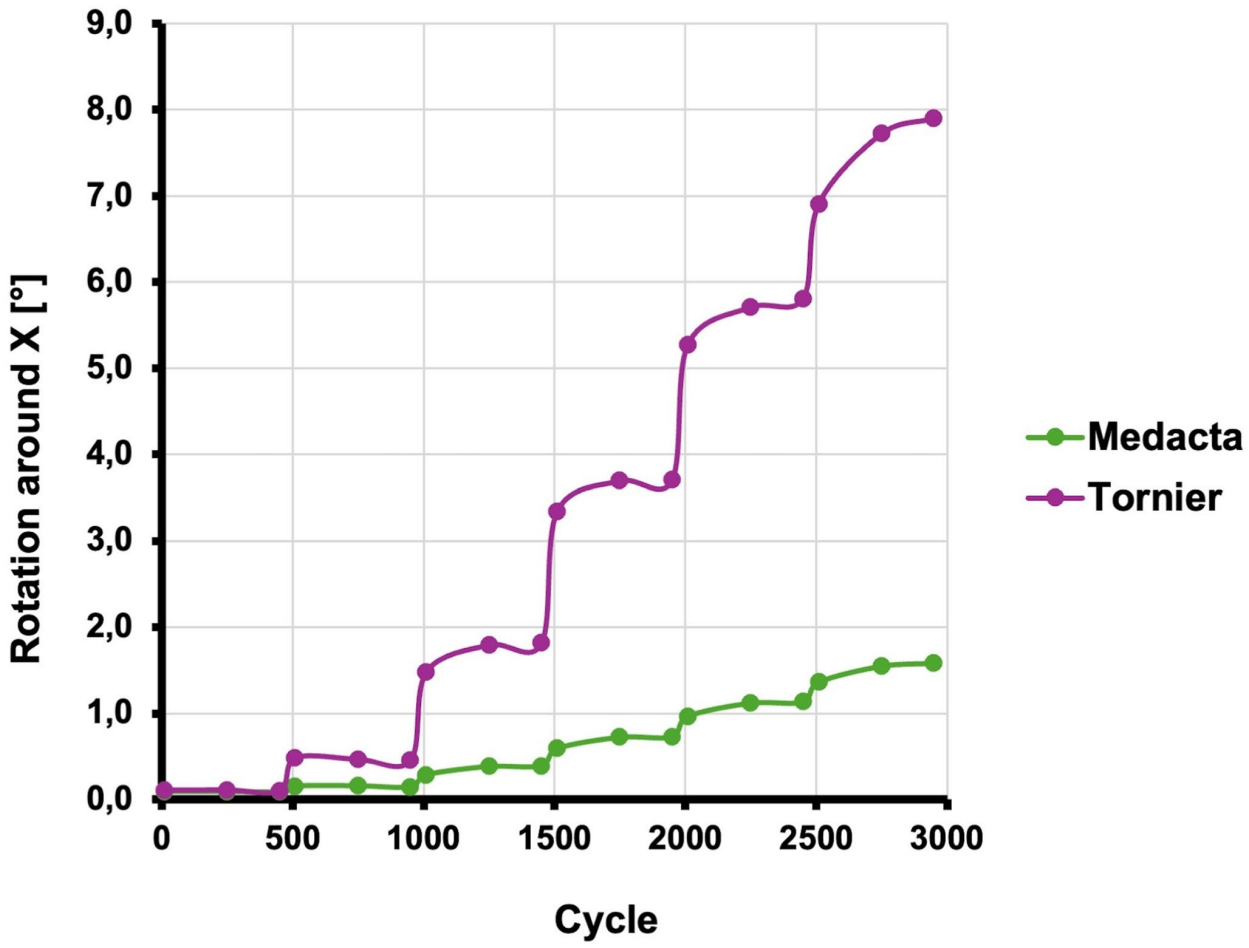
Relative rotation of two stem designs. Although no noticeable difference in relative rotation was noted in load levels one to three, the *Medacta short stem prosthesis* group (group B; green line) exhibited significantly greater rotational stability at load levels four (2.0 Nm), five (2.5 Nm), and six (3.0 Nm) (*p* = 0.047; *p* = 0.034; *p* = 0.016)

A significant difference was observed between the *Tornier Flex Shoulder System* group (group A) and the *Medacta short stem prosthesis* group (group B) in load level four (2.0 Nm) (t(4)=− 2.845; *p* = 0.047; d=− 1.41), five (2.5 Nm) (t(4)=− 3.152; *p* = 0.034; d=− 1.272) and six (3.0 Nm) (t(4)=− 4.028; *p* = 0.016; d=− 1.802) with the *Medacta short stem prosthesis* group (group B) demonstrating a significantly higher rotational stability. Again, the *Tornier Flex Shoulder System* group (group A) showed more variability (see Table 3 and Fig. 3).

**Table 3:** Relative rotation of two stem designs. Although no noticeable difference in relative rotation was noted in load levels one to three, the *Medacta short stem prosthesis* group (group B) exhibited significantly greater rotational stability at load levels four (2.0 Nm), five (2.5 Nm), and six (3.0 Nm) (*p* = 0.047; *p* = 0.034; *p* = 0.016). SD = standard deviation

Load Level	Tornier (Group A)	Medacta (Group B)	T(df)	Cohens‘d	*p*-Wert
1	0.107° (SD ± 0.02)	0.0916° (SD ± 0.01)	−1.558 (4)	−0.697	0.194
2	0.4702° (SD ± 0.34)	0.1532° (SD ± 0.09)	−1.872 (4)	−0.837	0.135
3	1.6974 (SD ± 1.00)	0.3534° (SD ± 0,49)	−2.635 (4)	−1.179	0.058
4	3.581° (SD ± 1.98)	0.683° (SD ± 1.17)	−2.845 (4)	−1.272	0.047
5	5.5954° (SD ± 2.69)	1.0746° (SD ± 1.93)	−3.152 (4)	−1.41	0.034
6	7.5102° (SD ± 2.60)	1.5008° (SD ± 2.63)	−4.028 (4)	−1.802	0.016

### Filling ratio

All specimens were included in the analysis. The mean metaphyseal filling ratio was 0.50 (range 0.43–0.56) in the *Tornier Flex Shoulder System* group (group A) and 0.63 (range: 0.57–0.70) in the *Medacta short stem prosthesis* group (group B) (Table 4).

**Table 4:** Filling ratio (FR) for both groups. The filling ratio was, both metaphyseal and diaphyseal, significantly higher in the *Medacta short stem prosthesis* group (group B). SD = standard deviation

	Tornier (Group A)	Medacta (Group B)	Cohen’s d	*p*-value
Metaphyseal FR	0.50 (SD ± 0.04)	0.63 (SD ± 0.05)	2.401	0.002
Diaphyseal FR	0.48 (SD ± 0.4)	0.66 (SD ± 0.04)	2.759	0.001

The mean diaphyseal filling ratio was 0.48 (range: 0.45–0.55) in the *Tornier Flex Shoulder System* group (group A) and 0.66 (range 0.62–0.71) in the *Medacta short stem prosthesis* group (group B). The filling ratio was, both metaphyseal (t(5) = 5.882; *p* = 0.002; d = 2.401) and diaphyseal (t(5) = 6.757; *p* = 0.001; d = 2.759), significantly higher in the *Medacta short stem prosthesis* group (group B).

## Discussion

The key finding of this study was the significant disparity in relative rotation observed between the cylindrical (group A) and the rectangular group (group B). Although no noticeable difference in relative rotation was noted in load levels one to three, the rectangular group (group B) exhibited significantly greater rotational stability at load levels four (2.0 Nm), five (2.5 Nm), and six (3.0 Nm) (*p* = 0.047; *p* = 0.034; *p* = 0.016).

On the other hand, the cylindrical group (group A) exhibited significantly increased levels of rotation, beginning at load level four (*p* = 0.047; *p* = 0.034; *p* = 0.016) and a greater rotational variability compared to the rectangular group (group B) across all loading levels.

To this date, there is little evidence that implant design has an impact on the long-term stability of shoulder arthroplasty. Existing basic considerations, however, derive from experiments on hip and knee arthroplasty and therefore it is common knowledge that a stable long-term osseointegration is achieved through a high primary stability, the avoidance of stress shielding and the prevention of implant migration [23]. Even stemless implants are increasingly considered for younger patients as they show lower short- to mid-term implant failure rates [5].

The present study focused on the subject of primary in-vitro stability imitating the immediate postoperative situation, which is, to our best knowledge, described for the first time for short stem arthroplasty.

A strong primary stability is essential for the successful integration of implants in the early post-operative period [23]. This primary stability is established during the implantation process, while secondary osseointegration occurs as the bone undergoes healing and remodeling [24]. Typically, this primary stability is achieved through a secure press-fit implantation during surgery [14].

This study therefore aimed to investigate whether design modifications, other than stem length, can impact the primary stability of short stem implants in vitro. The primary design difference between the cylindrical and the rectangular prosthesis is the stem’s geometry. Also, the filling ratio was notably higher in the rectangular group (*p* = 0.001), despite both systems being implanted by the same short-stem experienced shoulder surgeon who followed the manufacturers’ guidelines. Data by Barth et al. [2], who performed a finite element analysis to investigate the influence of proximal shaft geometry on torsional stability and stress distribution found that larger stem sizes exhibited better rotational stability and therefore support our findings.

However, these results should be interpreted with caution because larger stem sizes lead to higher filling ratios and higher filling ratios are associated with stress shielding [15]. Langohr et al. [10] assessed the effect of short-stem humeral component sizing on the appearance of humeral bone stress. Their results indicated that smaller short-stems are favorable in terms of stress shielding which goes in line with the findings by Denard et al. [4] and Raiss et al. [15]. The authors have therefore recommended no oversizing of humeral short stem implants [15, 19]. Further, Lacroix et al. emphasized in a finite element study on the influence of the geometry of the humeral head implant on cortical rim loading and therefore the stress shielding potential [9].

It is to be discussed if a surgeon, based on the current literature and findings of this study, should aim for a maximum stable primary fixation, which would be a press-fit implantation, to facilitate a secure secondary osseointegration. Or if a surgeon should elect for the first rotational stable stem within the cancellous bone to avoid oversizing and therefore stress shielding over time. A clear answer cannot be given at this point. However, initial high rates of stress shielding, associated with high filling ratios and cortical contact were observed in the first generation of the cylindrical *Tornier Flex Shoulder System* [17, 19]. In our own clinical procedure, passive elevation of the arm above 90° is restricted within the first two weeks and active assisted motion starts in week five after shoulder arthroplasty with the cylindrical stem design to allow the stem to grow into the proximal bone and achieve a stable osseointegration. The reduced primary stability of this stem implant does not seem to have an impact on the secondary stability to this date. However, more long-time studies and also long-time studies on other designs have to be awaited.

The variations identified in this basic experimental study suggest that each specific stem design should be independently monitored in clinical practice, and conclusions regarding long-term stability cannot be made solely based on stem length. The amount of stress shielding, for example, is not automatically reduced by the implantation of a shorter stem. Instead, proximal geometry, implantation technique, pre-operative planning, assessment of bone quality, modulus of elasticity and surface characteristics are all individual factors that have to be considered [23].

### Limitations

The study results are limited by a small number of cases. Due to the age of the body donors, the bone density is generally considered low. In the context of this experiment, primary stability was examined in the immediate postoperative situation, and no conclusions can be drawn regarding long-term osseointegration. Furthermore, a direct link from in vitro to in vivo should be interpreted with caution.

## Conclusion

The cylindrical stem design shows a higher relative rotation compared to the rectangular stem in vitro imitating the immediate postoperative situation, indicating that rotational stability might depend on the implant design. However, the mid- to first long-term rates of aseptic stem loosening for the cylindrical stem design are generally low. It is important to consider the sensible postoperative healing phase during postoperative rehabilitation, especially for cylindrical stem designs, to promote secondary osseointegration.

## Data Availability

No datasets were generated or analysed during the current study.
